# DMPA-SC stock: Cross-site trends by facility type

**DOI:** 10.1016/j.conx.2022.100075

**Published:** 2022-04-08

**Authors:** Sophia Magalona, Shannon N. Wood, Frederick Makumbi, Funmilola M. OlaOlorun, Elizabeth Omoluabi, Akilimali Z. Pierre, Georges Guiella, Jane Cover, Philip Anglewicz

**Affiliations:** a Department of Population Family and Reproductive Health, Johns Hopkins Bloomberg School of Public Health, Baltimore, MD, USA; b Department of Epidemiology and Biostatistics, School of Public Health, Makerere University College of Health Sciences, Kampala, Uganda; c Department of Community Medicine, University of Ibadan, Ibadan, Nigeria; d Statistics and Population Studies Department, University of the Western Cape, Bellville, South Africa; e Kinshasa School of Public Health, Kinshasa, Democratic Republic of the Congo; f Institut Supérieur des Sciences de la Population (ISSP/University of Ouagadougou), Ouagadougou, Burkina Faso; g PATH, Seattle, WA, USA

**Keywords:** DMPA-SC, Sayana press, Family planning, Reproductive health, Supply chain, Sub-Saharan Africa

## Abstract

**Objectives:**

To measure trends in the supply of DMPA-SC in public and private health facilities and compare with other prominent modern methods.

**Study design:**

We used repeated cross-sectional data from service-delivery-point surveys in six settings: Burkina Faso, Democratic Republic of Congo (Kinshasa and Kongo Central), Nigeria (Kano and Lagos), and Uganda, each with 3-5 rounds of data collected between 2016 and 2020. We analyzed trends in DMPA-SC availability using percent of service delivery points offering the method and percent experiencing stockouts; trends were compared with those for DMPA-IM, IUD, implants, and other short-acting methods, by facility type.

**Results:**

All settings showed increased offering of DMPA-SC over time for both private and public facilities. Larger proportions of public facilities provided DMPA-SC compared to private facilities (66%–97% vs 16%–50% by 2019–2020). DMPA-SC was provided by fewer facilities than DMPA-IM (90%–100% public, 34%–69% private by 2019–2020), but comparable to implants (83%–100% public, 15%–52% private by 2019–2020) and IUDs (55%–91% public, 0%–44% private by 2019–2020). Trends of DMPA-SC stock varied by setting, with more consistent stock available in private facilities in the DRC and in public facilities in Burkina Faso and Nigeria. Uganda showed decreasing stock in public facilities but increasing stock in private facilities.

**Conclusion:**

DMPA-SC availability has been increasing since its introduction in sub-Saharan Africa, yet significant gaps in stock exist. Countries should consider alternative distribution models to address these issues.

**Implications:**

Our findings may help inform countries about the need to monitor DMPA-SC availability and to consider solutions that ensure contraceptive options are available to women who need them and disruptions to contraceptive use are minimized.

## 1 Introduction

Subcutaneous depot medroxyprogesterone acetate (DMPA-SC), also known as Sayana^Ⓡ^ Press, is a relatively new injectable contraceptive method first introduced in sub-Saharan Africa (SSA) from 2014 to 2015. Its benefits include a delivery design that combines the drug and needle in a prefilled system, making it simple to administer and suitable for self-injection [1,2]. Given these attributes, it is not surprising that trends show increasing prevalence of DMPA-SC in many settings [3]. According to the DMPA-SC Access Collaborative—a project led by PATH and John Snow, Inc. that supports countries in the introduction and scale-up of the product—approximately 53 countries offer DMPA-SC as of 2021, and up to 33 are currently either piloting or scaling up DMPA-SC self-injection [4]. Many current DMPA-SC users are also first-time users of contraception, suggesting that DMPA-SC is reaching new populations, and can potentially increase overall modern contraceptive prevalence in some settings [5].

Despite its potential, research on DMPA-SC is limited, primarily due to the lack of representative data. Demographic and Health Surveys (DHS) do not differentiate between intramuscular or subcutaneous injectables. Routine information from the Health Management Information Systems (HMIS) or Logistics Management Information Systems (LMIS) are also lacking, with insufficient client characteristics, incomplete reporting from private facilities, and data aggregations that restrict client-level analysis [6,7]. The limited set of studies on supply-side factors mostly explore service provider-related characteristics and behaviors, with minimal or no data on method availability [8], [9], [10], [11]. The Performance Monitoring for Action (PMA) project collects national data on DMPA-SC from service delivery points and a representative sample of women, but the few publications to date have largely focused on user characteristics [3,5,12].

Understanding the availability of DMPA-SC provides rare supply side information to contextualize method use and shows whether advanced provision of DMPA-SC supplies for use in the future is feasible, given efforts advocating for it as a method of self-care. In addition, patterns of DMPA-SC use could be explained not just by user characteristics, but also availability in the health system. Diversification of service delivery channels is necessary to allow women a range of options in line with their preferences and lifestyles. Given the increasing popularity of DMPA-SC and the lack of routine data, monitoring DMPA-SC stock levels can help assess whether supply is keeping up with increased demand.

To fill this gap, we used facility-based data from Burkina Faso, Kinshasa and Kongo Central in the Democratic Republic of Congo (DRC), Kano and Lagos in Nigeria, and Uganda to examine changes in DMPA-SC availability in public and private health service delivery points between 2016 and 2020 and to compare these changes with trends observed for other prominent modern methods.

## 2 Methods

### 2.1 Study Overview

We used service-delivery-point data from the PMA project. Since 2013, PMA has collected representative data across SSA on key family planning indicators at the household and female levels and from select service delivery points. The data focus on the provision and quality of reproductive health services within the health service delivery point. DMPA-SC has been included since 2016. Further details on methodology can be obtained at www.pmadata.org and from Zimmerman et al. 2017 [13]. PMA received ethical approval from institutional review boards in each country.

### 2.2 Service-Delivery-Point Survey Administration

Eligible facility types were those that provide family planning in each setting. Types of service delivery points vary by country, and generally include health clinics, health centers, hospitals, pharmacies, and drug shops. They have different managing authorities, such as government, nongovernmental organizations, faith-based organizations, private sector organizations, and other institutions. Service delivery points managed by government are categorized as public facilities, whereas the rest are categorized as private facilities. The sampling approach aims to include approximately 3 private and 3 public service delivery points within or serving a census enumeration area (EA). For public facilities, PMA obtains a list of all public facilities in consultation with local health authorities and selects the tertiary, secondary, and primary facilities that serve the EA. To select private facilities, PMA conducts a mapping and listing of all facilities within the EA and randomly selects 3 for interview (or fewer, depending on the overall number in the EA). After identifying the service delivery points, a survey collecting data on contraceptive stock, cost, quality of services, and other related topics is administered.

### 2.3 Analytical sample

We included all PMA geographies that collected DMPA-SC data for at least 3 consecutive annual service-delivery-point surveys (Burkina Faso, Uganda, DRC, and Nigeria). Subnational trends were presented separately for DRC (Kinshasa and Kongo Central) and Nigeria (Kano and Lagos). Data were collected annually from 2016 to 2020. The number of rounds included in our analytical sample are as follows: Burkina Faso-5, DRC-Kinshasa-5, DRC-Kongo Central-4, Nigeria-Kano-3, Nigeria-Lagos-3, and Uganda-3 (Table 1).

**Table 1:** Six settings in sub-Saharan Africa with at least three data collection rounds between 2015 and 2020 included in DMPA-SC stock analysis.

	2015	2016				2017				2018				2019				2020	
SETTING	Q4	Q1	Q2	Q3	Q4	Q1	Q2	Q3	Q4	Q1	Q2	Q3	Q4	Q1	Q2	Q3	Q4	Q1	Q2
Burkina Faso		R3		R4			R5			R6			Phase 1						
DRC: Kinshasa	R4		R5				R6				R7				Phase 1				
DRC: Kongo Central				R5				R6				R7				Phase 1			
Nigeria: Kano							R4				R5						Phase 1		
Nigeria: Lagos							R4				R5						Phase 1		
Uganda							R5				R6				R6FU				

### 2.4 Measures

The key measures of interest were contraceptive method availability and stock status. Method availability was measured in PMA via multi-choice question: “Which of the following methods are provided to clients at this facility?” Interviewers then read a list of contraceptive methods including DMPA-SC aloud, and respondents confirmed whether each method was provided or not. To measure stock status, respondents were asked to confirm whether each product that they reported to provide was either in stock or out of stock. If a product was in stock, respondents were further asked “Has the [method] been out of stock at any time in the last 3 months?” Combining data from these 2 questions, stock status for each method was analyzed using 3, mutually exclusive categories: “currently in-stock,” if they had stock at the time of the survey and had not experienced a stockout over the previous 3 months; “currently in-stock but had a recent stockout” if they had stock at the time of the survey, but experienced a recent stockout; or “currently out-of-stock” if they did not have stock at the time of the survey.

DMPA-SC trends were compared to other modern methods, namely DMPA-IM, intrauterine devices (IUD), implants, and other short-acting methods (oral pills, male and female condoms, emergency contraception, standard days/cycle beads, diaphragm, and foam/jelly). Service delivery points were coded as providing other short-acting methods if they reported providing at least one of the included methods and coded as in stock if they had any in stock. Analyses were stratified by public and private managing authority. Number of service delivery points per facility type, by round of data collection and setting are outlined in Appendix 1. On average across all time points, higher proportions of public than private facilities were observed in Burkina Faso, Nigeria, and Uganda (ranging from 60% to 85%), whereas in DRC it was the opposite (13% in Kinshasa and 49% in Kongo Central; percentages not shown).

### 2.5 Analytic methods

For each setting, we tabulated the percentage of service delivery points that provided DMPA-SC over the period from 2016 to 2020 by facility type, and then compared this to provision trends observed for other modern methods over the same period by facility type. Next, within each setting, we presented trends of DMPA-SC stock status by facility type, limiting our analysis to service delivery points that reported providing DMPA-SC. All analyses were conducted using Stata 16.1.

## 3 Results

### 3.1 Trends in DMPA-SC provision

**Fig. 1:**
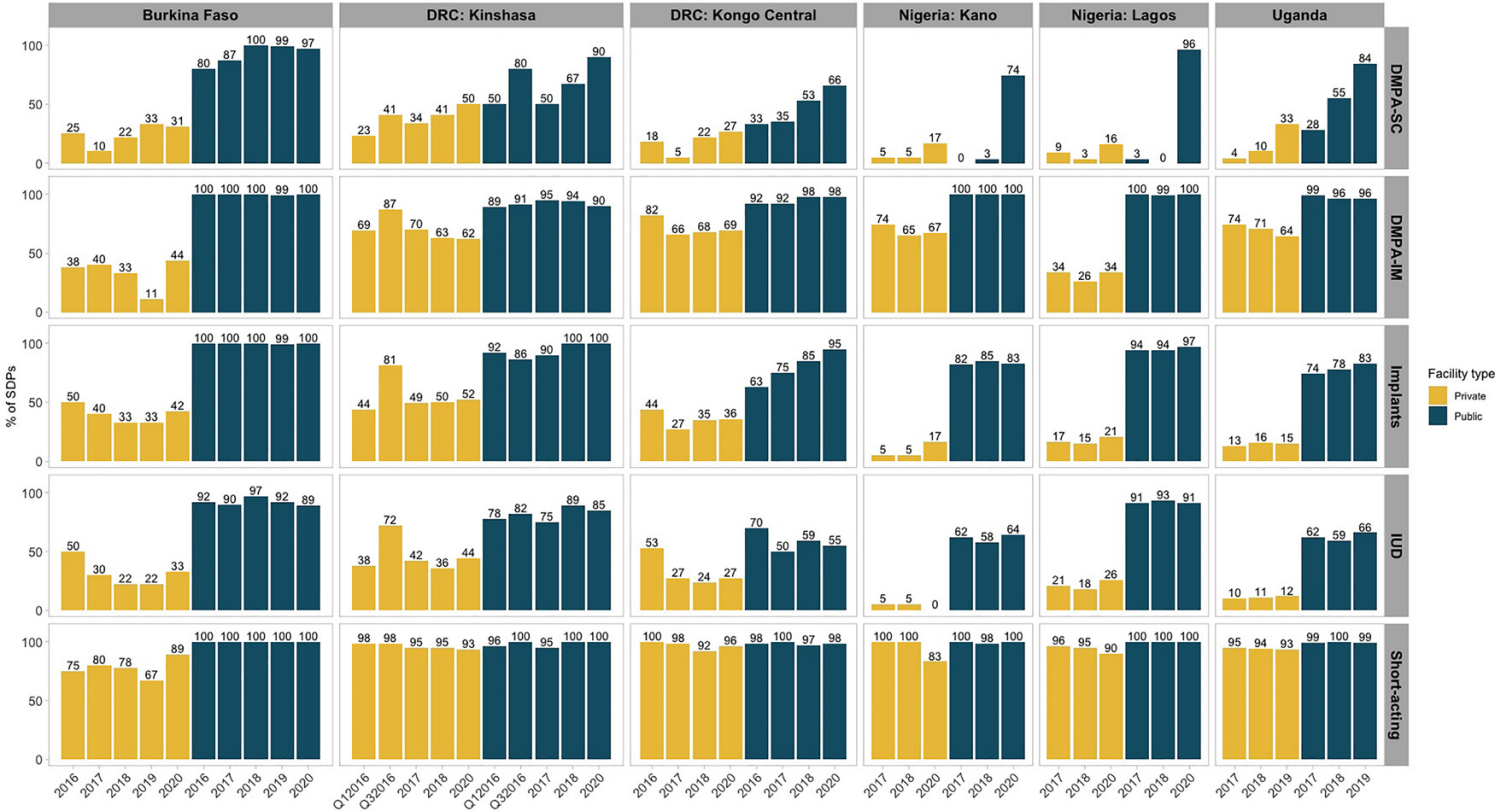
Percentage of service delivery points that provide DMPA-SC and other modern methods by facility.

All settings generally showed increasing provision of DMPA-SC for both private and public facilities (Fig. 1). More public facilities provided DMPA-SC compared to private facilities, and this difference was consistent over time. By latest survey round, only 16% to 50% of private facilities provided DMPA-SC, compared to 66% to 97% of public facilities across settings.

Most settings saw steady increases in DMPA-SC provision over time; however, in Kano and Lagos, provision remained low in both public and private facilities until 2020, when we see a sharp increase in public facility provision (0%–74% Kano; 3%–96% Lagos). Similar increases were not observed in private facilities, where provision remained low (17% Kano; 16% Lagos).

Burkina Faso showed consistently high DMPA-SC provision in public facilities, increasing from 80% in 2016 to 100% by 2018 and remaining around this level until 2020. While Uganda's public provision reached 84% in 2019, this increase was gradual. Comparatively, private facility provision was approximately 30% in both settings.

In the DRC, DMPA-SC provision increased steadily over time in Kongo Central for both private and public facilities; however, by 2020, only 27% of private facilities and 66% of public facilities provided the method. These levels were lower than those observed in Kinshasa, where 50% of private facilities and 90% of public facilities provided DMPA-SC by 2020.

### 3.2 Trends in provision of other modern methods

#### 3.2.1 DMPA-IM

Similar to DMPA-SC, provision of DMPA-IM was higher in public facilities than private facilities across all settings (Fig. 1). DMPA-IM was consistently provided in at least 89% of public facilities in any given year. Conversely, provision of DMPA-IM in private facilities varied substantially across settings.

#### 3.2.2 Other short-acting methods

Other short-acting methods were provided by almost all public and private facilities, except in Burkina Faso (75%–89% of private facilities; Fig. 1).

#### 3.2.3 Implants

Implants were provided nearly universally in public facilities and consistently at the same level within each setting over time, except for Kongo Central where provision increased by 32% in 4 years, and Uganda with a modest increase of 9% over 3 years (Fig. 1). In the private sector, a lower proportion of facilities provided implants, with the lowest levels in Nigeria and Uganda.

#### 3.2.4 IUD

IUD offerings in the public sector were generally lower than other methods, ranging from 55% in Kongo Central to 91% in Lagos (Fig. 1). These proportions were only slightly lower than DMPA-SC by 2019–2020. Private sector provision was particularly low in Uganda (12%) and Kano (0%).

### 3.3 DMPA-SC stock status trends

**Fig. 2:**
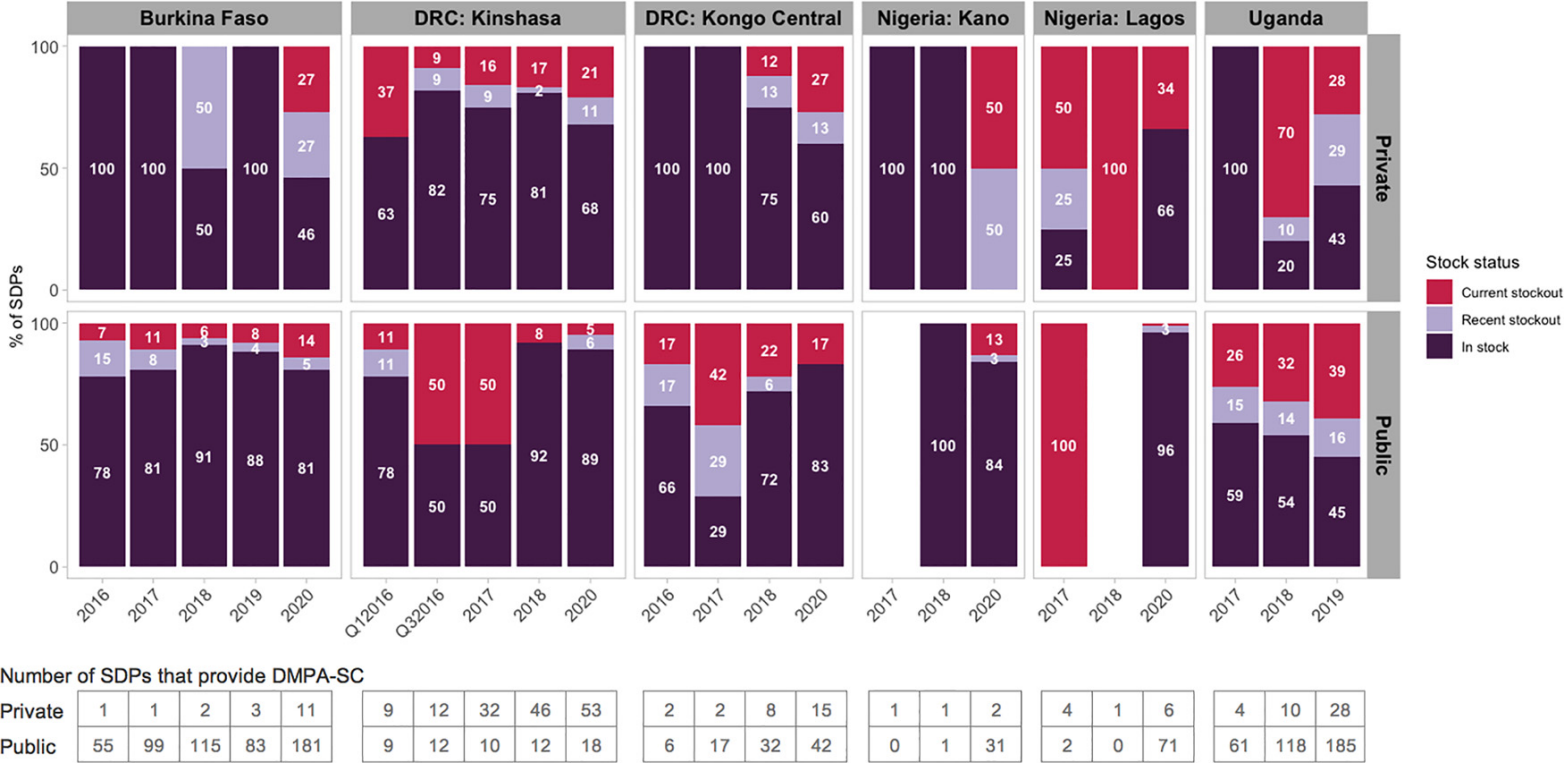
DMPA-SC stock status in public and private service delivery points.

Trends of DMPA-SC stock availability varied substantially by setting (Fig. 2). In Burkina Faso, where DMPA-SC was provided in a majority of public facilities, the product was consistently in stock in over 78% of facilities every year. Public facilities with current or recent stockouts ranged from 9% to 22% from 2016 to 2020. In private facilities, availability was inconsistent, with 100% availability in some years (2016, 2017, and 2019) to 50% or less in others (2018 and 2020). Notably, 27% of private facilities experienced a current stock-out and another 27% reported a recent stock-out by 2020.

In Kinshasa and Kongo Central, private facilities showed more consistent availability of DMPA-SC over time (60%–100% in stock). Public facilities showed a decrease in DMPA-SC availability between 2016and 2017, but rebounded thereafter. By 2020 availability of DMPA-SC in public facilities was slightly higher than in private facilities, with 32% to 40% of private facilities experiencing a recent or current stockout.

In Kano, stock was 100% available in the 3 public and private facilities that offered DMPA-SC. By 2020, availability maintained at 84% in public facilities; however, the 2 private facilities experienced a recent or current stockout. Similar stock trends were not viewed in Lagos. The few public and private facilities that offered DMPA-SC in 2017 and 2018 almost always experienced a recent or current stockout. By 2020, availability increased to 96% in public facilities and 66% in private facilities.

In Uganda, DMPA-SC stock availability decreased over time in both public and private facilities. In public facilities, current stockouts increased from 26% in 2017 to 39% in 2019; whereas in private facilities, recent stockouts increased from 10% to 29%, while current stockouts were cut by more than half from 2018 to 2019.

## 4 Discussion

This is the first study to describe trends of DMPA-SC provision and stock availability and compare with other contraceptive products at public and private facilities across multiple country settings. Specifically, we examined DMPA-SC supply side trends, using data from 6 settings. Results showed that an increasing number of facilities provided DMPA-SC over time in both the public and private sectors, and across all settings. More public facilities provided the method than private, but this difference was not unique to DMPA-SC. This pattern was also observed for DMPA-IM and long-acting methods.

A strength of this study is its use of facility data at multiple time points and settings. The DHS Service Provision Assessments (SPA) only provide a snapshot of contraceptive supply and availability at irregular intervals and for a limited number of countries [14]. Among the 4 countries included in our analysis, the SPA has only been completed in Uganda in 2007 and the DRC in 2017–2018. An increasing number of countries use information systems such as HMIS or LMIS to monitor consumption over time. However, in the HMIS, data on contraceptive stock is limited, with some only capturing information for a tracer commodity (DMPA-IM in Uganda, e.g.) instead of all methods [15]. Our data also includes both public and private sectors. The HMIS and LMIS often do not capture data from private sources, whereas SPAs do not include pharmacies in their sample.

Our study observed supply side trends that are consistent with increasing DMPA-SC prevalence across SSA [3]. Results are also consistent with previous research on other modern methods describing the wide variation in contraceptive stock availability across countries, methods, and sectors [16], [17], [18], [19]. This variation may be due to varying supply chain models and their respective challenges in these different settings. Burkina Faso and DRC utilize a pull distribution system in which procurement is decentralized and contraceptive orders are decided by lower levels of the system based on consumption data. In contrast, Nigeria uses a push system in which resupply levels are decided at central hubs informed by general consumption data, population estimates, and stock availability at the central level [19]. Nigeria also has a task-shifting and task-sharing policy that allows trained community health workers to administer DMPA-SC. Uganda, on the other hand, is in the process of transitioning from a hybrid model to a full pull system. Divergence by setting in provision and stock trends over time may also be due to country-level priorities in DMPA-SC roll-out. For example, in the first years of introduction, Uganda limited provision via community health workers rather than engaging facility-level providers. Community health workers lack adequate resources and support from the health system, often citing stockouts as a problem in performing their job [20]. In addition, we observed consistently higher availability of injectables, IUD, and implants in public facilities than private facilities similar to other studies [16,19], especially in most recent years. For IUDs and implants, the difference could be due to the training and equipment required for insertion, which may be more variable among private facilities due to the inclusion of a range of facility types, from hospitals to drug shops. For DMPA-SC, the difference may be due to the pattern of introduction, such as the case of Burkina Faso where the product was first introduced in public facilities, or to the strict regulatory environment, which may limit who can sell the product or impose rebranding restrictions [21].

Despite its relatively recent introduction, DMPA-SC provision has reached the same degree of availability as implants and IUDs. It can potentially surpass them to reach the same level as DMPA-IM, as previous studies have described how short-acting reversible methods, including injectables, are more available than long-acting ones, such as implants and IUDs [15,17,19]. Short-acting methods may be more appealing to younger women who want to delay or space childbirth, rather than limit. Indeed, some studies have shown that providers and clients may prefer DMPA-SC over DMPA-IM [2,22]. To embrace client-centered care family planning programs should endeavor to provide methods that best meet client needs and preferences.

This research is not without limitations. Given annual data collection, we could not detect potential changes in stock patterns at shorter time intervals. Previous work found variation in contraceptive availability in 4 African countries using data collected quarterly in only 2 years [19]. Our data are also limited to only up to 3 private and up to 3 public service delivery points, and for some data collection rounds stock status was available for only a small number of facilities. Another limitation is that while we defined facilities based on managing authority, in some settings, like the Democratic Republic of Congo, the delineation between private and public facilities is not clear, with some government facilities receiving support from private and not-for-profit institutions and vice versa [23]. Per PMA protocol, the public categorization takes precedence in such cases. Stockouts were also measured only for the day of the survey or at any time in the 3 months prior, with no information on duration. Finally, unlike the female data, the service-delivery-point data from PMA are not designed to be representative of all service delivery points.

Overall, despite the increasing availability of DMPA-SC, availability varies by facility type and across settings. Persistent stockouts and limited method options can disrupt contraceptive use and increase discontinuation [24]. Alternative distribution models and a generic product offering may address these issues and potentially increase the availability of DMPA-SC. Ultimately, countries should consider solutions that best address their context-specific challenges with the aim of ensuring that women have a range of contraceptive options available to them so they can achieve their fertility goals.

## Figures and Tables

**Table:** 

	Round	Number of facilities		
		Private	Public	Total
Burkina Faso	2016	22	110	132
	2017	15	114	129
	2018	15	115	130
	2019	12	84	96
	2020	48	186	234
DRC: Kinshasa	Q1 2016	197	31	228
	Q3 2016	147	24	171
	2017	151	24	175
	2018	165	21	186
	2020	177	26	203
DRC: Kongo Central	2016	47	55	102
	2017	60	55	115
	2018	60	64	124
	2020	85	68	153
Nigeria: Kano	2017	23	46	69
	2018	25	45	70
	2020	20	45	65
Nigeria: Lagos	2017	50	67	117
	2018	47	73	120
	2020	49	78	127
Uganda	2017	116	219	335
	2018	126	216	342
	2019	108	220	328
